# Synthetic single cell RNA sequencing data from small pilot studies using deep generative models

**DOI:** 10.1038/s41598-021-88875-4

**Published:** 2021-04-30

**Authors:** Martin Treppner, Adrián Salas-Bastos, Moritz Hess, Stefan Lenz, Tanja Vogel, Harald Binder

**Affiliations:** 1 Institute of Medical Biometry and Statistics, Faculty of Medicine and Medical Center, University of Freiburg, 79104 Freiburg, Germany; 2 Freiburg Center for Data Analysis and Modeling, University of Freiburg, 79104 Freiburg, Germany; 3 Department of Molecular Embryology, Medical Faculty, Institute of Anatomy and Cell Biology, University of Freiburg, 79104 Freiburg, Germany; 4 Faculty of Biology, University of Freiburg, Freiburg, Germany; 5 Center for Basics in NeuroModulation (NeuroModul Basics), University of Freiburg, 79104 Freiburg, Germany; 6 Freiburg Institute for Advanced Studies (FRIAS), University of Freiburg, Freiburg, Germany

**Keywords:** Computational models, Machine learning, Statistical methods

## Abstract

Deep generative models, such as variational autoencoders (VAEs) or deep Boltzmann machines (DBMs), can generate an arbitrary number of synthetic observations after being trained on an initial set of samples. This has mainly been investigated for imaging data but could also be useful for single-cell transcriptomics (scRNA-seq). A small pilot study could be used for planning a full-scale experiment by investigating planned analysis strategies on synthetic data with different sample sizes. It is unclear whether synthetic observations generated based on a small scRNA-seq dataset reflect the properties relevant for subsequent data analysis steps. We specifically investigated two deep generative modeling approaches, VAEs and DBMs. First, we considered single-cell variational inference (scVI) in two variants, generating samples from the posterior distribution, the standard approach, or the prior distribution. Second, we propose single-cell deep Boltzmann machines (scDBMs). When considering the similarity of clustering results on synthetic data to ground-truth clustering, we find that the $scVI_{posterior}$ variant resulted in high variability, most likely due to amplifying artifacts of small datasets. All approaches showed mixed results for cell types with different abundance by overrepresenting highly abundant cell types and missing less abundant cell types. With increasing pilot dataset sizes, the proportions of the cells in each cluster became more similar to that of ground-truth data. We also showed that all approaches learn the univariate distribution of most genes, but problems occurred with bimodality. Across all analyses, in comparing 10$\times$ Genomics and Smart-seq2 technologies, we could show that for 10$\times$ datasets, which have higher sparsity, it is more challenging to make inference from small to larger datasets. Overall, the results show that generative deep learning approaches might be valuable for supporting the design of scRNA-seq experiments.

## Introduction

Deep generative models, such as variational autoencoders (VAEs)^1,2^ or deep Boltzmann machines (DBMs)^3^, can learn the joint distribution of various types of data, and impressive results have been obtained, e.g., for generating super-resolution images in microscopy^4^ and more generally for imputation^5,6^. This raises the question of whether such techniques could also be trained on data with a rather small number of samples, e.g., obtained from pilot experiments, for subsequently generating larger synthetic datasets. Such synthetic observations could inform the design of single-cell RNA sequencing (scRNA-seq) experiments by exploring planned subsequent analysis steps, such as clustering, on synthetic datasets of different sizes.

ScRNA-seq experiments result in data reflecting gene expressions for individual cells in tissues, leading to an improved understanding of cell-type composition. Due to the underlying complexity, deep generative approaches are increasingly used to investigate the structure of scRNA-seq data by learning a low-dimensional latent representation of gene expression within cells. Often, the focus of these applications is on exploring latent features—representing cell types—after which they are used for clustering, imputation, or differential expression analysis^6–8^. As indicated, another interesting property of these generative approaches is that they can provide synthetic data once trained on some dataset. However, the quality of the data generated from these models is challenging to evaluate and requires cautious examination, depending on the field of application and the research question^9^. In the following, we investigate the quality of synthetic data from generative models and illustrate various obstacles for its application based on an example of the design of scRNA-seq experiments.

Single-cell experiments are often costly and time-consuming; hence, it is crucial to plan these experiments carefully to avoid wasting resources. Based on small pilot experiments, deep learning models could generate synthetic data to make judgments about potentially larger experiments, thus offering the possibility of gaining confidence in planning. The apparent advantage to this would be that scientists could generate a practically infinite amount of synthetic data, preventing experiments from being too small or too large. Moreover, synthetic data can also be valuable for experimental design by investigating in-silico generated cell perturbations^10^.

As the experimental design of scRNA-seq studies is often based on simulations^11–15^, synthetic data could be useful, e.g., when training a generative approach on some pilot data. Sampling from latent representations of generative models then allows for generating in-silico expression patterns, ideally reflecting the most important patterns from the pilot data, and can subsequently be utilized for planning experiments. More specifically, researchers would specify different numbers of cells to be simulated, then apply downstream analyses to the simulated data, after which they evaluate the number of cells needed for detecting patterns of interest, such as clusters comprising rare cell types.

**Figure 1:**
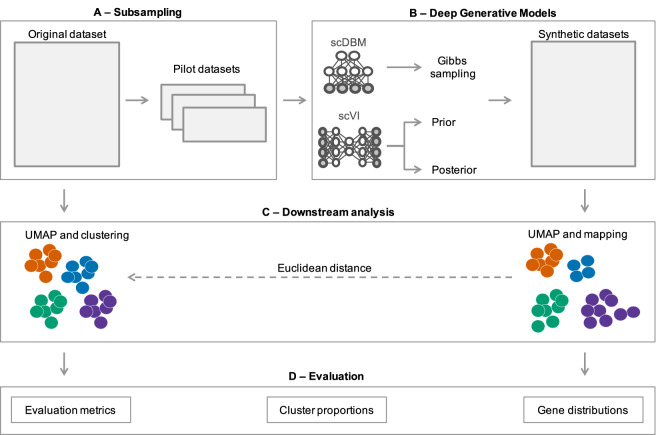
Design for evaluating the performance of deep generative models with small pilot datasets: (**A**) Take a sub-sample from an original dataset to obtain pilot data with known ground truth. (**B**) Train the deep generative approaches on the pilot dataset and generate synthetic data in the original data size. (**C**) Apply dimensionality reduction with UMAP and Seurat clustering to the original data and map each synthetic observation to the closest observation from the original data, thus getting a cluster assignment. (**D**) Evaluate the quality of synthetic samples based on the Davies–Bouldin index, adjusted Rand index, cluster proportions, and distributions per gene. The complete analysis is performed for different sizes of pilot datasets (384, 768, 1152, 1536, 1920, and 2304 cells) and repeated 30 times for each size.

To investigate the authenticity of the synthetic data using experimental design as an example, we follow the procedure shown in Fig. 1. First, we extract small pilot datasets from the larger original data by random sub-sampling, where each cell is drawn with equal probability (Fig. 1A). Next, we train the deep generative models on the sub-sampled pilot datasets and generate synthetic data in the size of the original study (Fig. 1B). We apply downstream analyses to both the original data and the synthetic data (Fig. 1C), after which we examine the quality of the synthetic observations using various evaluation approaches (Fig. 1D). We use the data labels resulting from the clustering of the original data as a reference to assume as little prior knowledge as possible about the sample in question. For example, if we were to use existing cell type labels, we could not rule out that the labels were created based on other experimental methods that are a bad fit for the current analysis. Since we assume that no clear information about possible cell types is available when planning an experiment, we avoid including information that would not be available in a realistic application setting.

We specifically selected three evaluation methods to investigate the extent to which synthetic data from the models considered can help recapitulate the multivariate structure of gene expression data. This is particularly relevant for clustering, and consequently, for the discovery of cell types. Accordingly, we examine both internal and external clustering evaluation metrics. More specifically, we chose the Davies–Bouldin index because it provides a value for the appropriateness of data partitions. Suppose synthetic data based on a small sub-sample of the original dataset yields similarly small values for the Davies–Bouldin index (DBI). In that case, we can assume that this clustering is also of similar quality, allowing the DBI of the clustering partition on the original data to be used as a benchmark. The DBI could be used to identify a saturation point in the number of generated cells beyond which the DBI does not substantially improve. Also, scientists can use the variability of the DBI across multiple sub-sampling iterations to infer an appropriate sample size, as lower variability indicates a more stable clustering solution. Furthermore, we use the adjusted Rand index (ARI) as an external evaluation criterion. The ARI gives us information about the similarity of two clusterings based on the original data labels. As a further criterion, we examine the cluster proportions to determine at which cluster size the methods encounter problems in recognizing and generating cells from this cluster. We use the univariate distributions of gene expression as another measure since the assignment of similar cells to a cell type is typically performed based on marker genes and may be of importance for discovering cell types.

While VAEs have already been proposed for scRNA-seq data^7^, DBMs still need to be adapted. We show how this can be achieved using a negative binomial distribution and incorporating a regularized Fisher scoring algorithm to estimate the inverse dispersion parameter. We chose DBMs because synthetic observations are generated by Gibbs sampling, which has theoretical properties that are potentially advantageous for working with smaller sample sizes than variational inference in VAEs^16,17^. Since we use the single-cell variational inference (scVI) approach in reduced form, i.e., we do not use batch correction or zero-inflation, the method is comparable to a vanilla VAE with a negative binomial loss function, except for the estimation of the size factors for normalization.

VAEs reconstruct their input through a bottleneck layer that corresponds to a low-dimensional latent representation. They offer two ways of generating samples from the latent representation. Most commonly, samples are generated from the posterior, which is the latent variables’ probability given the original data. In a pilot study setting, this will typically mean that multiple copies of the original observations have to be used to obtain a larger synthetic dataset. This might lead to an amplification of sampling bias, as patterns or random fluctuations from single cells could be over-emphasized. In contrast, sampling from the prior might produce samples from a diverse region of the latent space. In our evaluation together with DBMs, we therefore not only investigate the performance of VAEs when feeding in the original data multiple times for obtaining a larger number of cells but also when sampling directly from the prior, which has—to our knowledge—not been considered in the scRNA-seq literature so far.

Variational autoencoders are already widely established in application to scRNA-seq data. Researchers have developed many models to visualize scRNA-seq data based on the VAE architecture^18,19^. The optimal architecture for variational autoencoders with application to scRNA-seq data has also been investigated by comparing different count likelihood functions. The authors also propose an adapted architecture to include clustering information into the latent space^20^. Furthermore, VAEs were used to generate previously unseen responses to single-cell perturbations based on variables learned in the latent space^10^. scVI was one of the first fully-integrated models for the use of VAEs in application to scRNA-seq data. It offers the possibility to learn the scRNA-seq data using the negative binomial distribution and add a zero-inflation term. It is useful for many applications in scRNA-seq data analysis and is regularly extended to other omics technologies and applications^21–24^. Because of its widespread use, it is interesting to examine this model for settings with small sample sizes.

## Results

To examine the quality of the data generated by scVI and single-cell DBMs (scDBMs), we used the example of designing a scRNA-seq experiment. By mimicking a situation where we want to plan an experiment using a pilot study with a small number of cells, we investigated the impact of varying amounts of cells and generative approaches on the clustering performance, measured by the DBI. We took 30 sub-samples of 384, 768, 1152, 1536, 1920, and 2304 cells of the original dataset, trained the scDBM and scVI on these sub-samples, and generated synthetic data. More precisely, we sampled from the scDBM using Gibbs sampling and from scVI using the prior and posterior distribution, respectively. The sub-samples’ size is based on the number of cells that can be captured by a 384-well plate, which allows us to get an indication of the required number of plates for a sequencing experiment. We then applied UMAP and acquired the cluster labels by mapping the synthetic observations to the original data based on the Euclidean distance in UMAP space (Fig. 1C).

**Figure 2:**
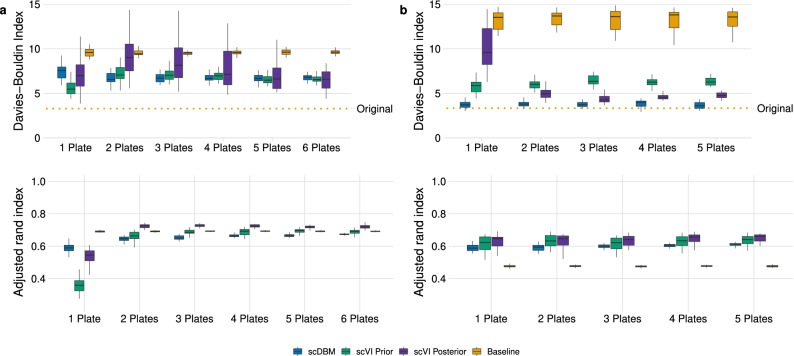
Davies-Bouldin index (top) and adjusted Rand index (bottom), indicating the quality of synthetic data generated by scDBM, scVI (prior and posterior sampling), and a baseline from pilot data of different sizes for *PBMC*4*k* (**a**) and *Segerstolpe* (**b**). Each boxplot represents 30 sub-samples from the original data (lower and upper hinges correspond to the 25th and 75th percentiles). The orange dotted line indicates the reference DBI for the Seurat clustering on the original data.

We have also added a performance baseline, which mainly provides an upper bound for the sampling bias. We generated negative binomial noise by randomly drawing the scale and shape parameters of a gamma distribution from Uniform(0, 5) and Uniform(0, 10), respectively. We used the values drawn for each cell as rate parameters in the Poisson distribution and added the resulting values to the sub-sampled pilot data. To be more precise, we have merged several noisy pilot datasets to achieve the original dataset’s size. For example, for a pilot dataset with 384 cells and an original dataset of 3840 cells, we replicated the noisy dataset ten times and then merged them. One would expect the DBI (see “Methods”) to be very high in a scenario with small pilot datasets since potential artifacts could be strongly amplified.

The results show that $scVI_{posterior}$ exhibits high variability, especially with small datasets. In contrast, the variability for scDBM and $scVI_{prior}$ is much lower. Regarding the variability, and thus the dependence of the models on the representativeness of the pilot data, we found similar results in other datasets (Supplementary Fig. 1, 2). With its high variability and some extreme outliers, $scVI_{posterior}$ sometimes even leads to worse results than our simple baseline, which is based solely on noisy pilot datasets (Fig. 2a,b, top). These findings support our hypothesis that posterior sampling in scVI leads to an amplification of the sampling bias when drawing conclusions for larger datasets based on small pilot studies. Furthermore, sparsity seems to impact the training and quality of the generated samples significantly. Looking at the *Segerstolpe* data, which were generated with the Smart-seq2 protocol and therefore exhibit less sparsity, the DBIs of all models show less variability and are closer to the DBI of the original data (Fig. 2b, top).

We noticed that, for the sparser datasets, the DBI was lowest for the smallest sample size, after which the DBI for two plates showed an increase for scVI (Fig. 2a, top). A possible explanation for this behavior would be that with only 384 cells drawn, some clusters are rarely or not at all represented by the sample. Since the deep generative models have a tendency to sparsity in the lower-dimensional latent space, they learn the structure with fewer clusters than they have in the original dataset. When doubling the sample size from one plate to two plates, some cells seem to be found for each cluster, but the number of cells does not seem sufficient for the models to properly learn the structure. This leads to an increase in the DBI. If the number of cases is further increased, the number of cells per cluster will also increase, and models will resolve these clusters more easily.

The ARI is low in particular for smaller datasets in the sparse *PBMC*4*k* data but stabilizes at a high level for as few as two plates. This can again be explained by the many zeros in the data since the models then effectively have fewer observations to learn the corresponding patterns (Fig. 2a, bottom). However, this is not particularly helpful in situations where we hope for a certain generalizability, i.e. from small to larger datasets.

**Table 1:** Mean of absolute differences in the number of cells across all clusters.

	Number of plates					
	1 Plate	2 Plates	3 Plates	4 Plates	5 Plates	6 Plates
**PBMC4k**						
scDBM	2691	2017	1910	1776	1737	1624
scVI prior	6567	4565	4352	4249	4205	4196
scVI posterior	5037	2965	2711	2599	2709	2513
**Segerstolpe**						
scDBM	1037	925	887	889	864	–
scVI prior	1273	1228	1108	972	857	–
scVI posterior	1293	1163	1122	856	645	–

Next, we inspected whether the models accurately estimated the proportions of cells per cluster to uncover heterogeneity and subpopulation frequencies. We calculated the mean of the absolute differences between the number of cells in the original dataset and the respective synthetic datasets across all clusters. With increasing sample size, the differences between original and synthetic data become smaller. However, there are large differences in the absolute values between the *PBMC*4*k* and the *Segerstolpe* data. This can again be explained by the less accurate estimates of the models with sparse data, where the estimates for the *Segerstolpe* dataset perform better (Table 1). This indicates that larger datasets might be needed to properly generate synthetic observations with adequate cluster proportions.

**Figure 3:**
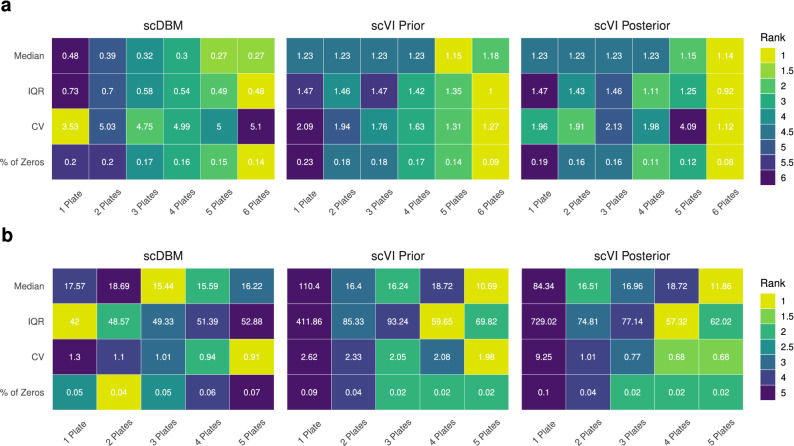
Mean absolute deviation for various descriptive statistics across all models and sample sizes for the *PBMC*4*k* dataset (**a**) and the *Segerstolpe* dataset (**b**). Color coding indicates ranks among sample sizes.

Furthermore, we calculated the mean absolute deviation (MAD) for each gene between the synthetically generated data and the original data for a range of metrics. More specifically, we compare medians, interquartile ranges, coefficients of variation, and the proportion of zero counts. In the *PBMC*4*k* dataset, it is apparent that the scDBM more accurately estimates medians and interquartile ranges across all sample sizes. For the coefficient of variation, the scDBM shows mixed results, whereas the VAE-based models perform better. All models show improved performance concerning the proportion of zero counts as sample size increases (Fig. 3a). A similar picture emerges for the *Segerstolpe* dataset, with scDBMs showing promising results for smaller sample sizes. However, the scVI variants improve their performance concerning the descriptive statistics quickly with increasing sample size (Fig. 3b). We also inspected the marginal distributions of several exemplary genes in samples from scVI and scDBM and compared them with the distributions in the original data (Supplementary Figs. 3 and 4). We observed that the synthetic data generated from the scDBM trained on one plate matches the true distribution of many genes rather well but tends to underestimate expression counts. In contrast, $scVI_{prior}$ and $scVI_{posterior}$ tend to overestimate expression in many genes. All methods frequently exhibit difficulties with bimodality, as can be seen in, e.g., CD74 of the *PBMC*4*k* dataset (Supplementary Fig. 3). If the models cannot learn this bimodality, false negatives may occur when testing differential expression between clusters. This is to be expected for small sample sizes, as the models may not have enough power to detect the two or multiple modes in the distribution of a gene. Compared to the sparse 10$\times$ genomics data, the scDBM on the *Segerstolpe* data exhibits fewer problems in estimating the marginal distributions. This might again be due to the lower sparsity, which seems to allow more realistic estimates at small sample sizes (Supplementary Fig. 4). When looking at the UMAP representations for an example run of the models on the *Segerstolpe* dataset, we can see similar patterns as for the DBI and ARI. All models have problems detecting smaller clusters at smaller sample sizes, but the performance of $scVI_{posterior}$ improves drastically with increasing sample size (Supplementary Fig. 7).

To ensure that the models we examined actually generate realistic data while not imputing zeros we inspected all models on a dataset with artificially added zeros. To this end, we first considered the proportion of zeros per gene in the original *Segerstolpe* dataset across all models, and second, we set 20$\%$ of the expression values in the *Segerstolpe* dataset to zero. We were able to show that all models also learn the *Segerstolpe* data with artificially added zeros properly without imputing the omitted expression values (Supplementary Fig. 5).

## Discussion

In this paper, we have investigated the quality of synthetic scRNA-seq data from deep generative models. We looked at situations where we want to draw conclusions from small amounts of data to larger, ground-truth data. This might be relevant, e.g., when planning single-cell RNA-sequencing experiments. To investigate the quality of the generated data, we have relied on three approaches. We used the DBI and ARI to compare the clusterings’ quality on the synthetic data with the original data. We also looked at how the different models behave in response to varying cluster sizes, and finally, we examined the synthetic data using descriptive statistics of univariate gene distributions.

We sub-sampled parts of the original dataset to mimic a pilot data scenario. Next, we trained the deep generative models on these sub-samples and generated synthetic observations in the original data size. For this, we used scDBM and scVI, where we draw samples from both the prior and posterior distribution for the latter model. In particular, when looking at small datasets, which may be subject to sampling artifacts, it is advisable to draw from the prior distribution, instead of the posterior, in scVI. Furthermore, in such scenarios, Markov chain Monte Carlo methods might have an advantage over variational inference, which is mainly reflected in the lower variability of scDBM. The results show that larger datasets might be necessary to generate synthetic data with proper cluster proportions.

We rely on the assumption that synthetic cells mapped to the original data are also mapped to the correct clusters. This assumption allows us to investigate whether a hypothetical cluster solution underlying the original data could be clearly seen from a certain number of cells. If some of the synthetic cells are mapped to the wrong clusters, this will typically mean that separation of the clusters is underestimated, i.e., our approach errs on the conservative side with respect to the required number of cells.

If conclusions are drawn from a small sample to a larger dataset, it is important that the pilot sample is representative. In the example of planning an experiment, the pilot study sample may subsequently be included in the main study. Still, care should be taken to ensure that the experimental settings are not changed to ensure representativeness^25^. Studies on the generalization ability of deep generative models already exist^26^, but they have not yet been extended to the application of single-cell transcriptomics data. Doing so is outside the scope of this paper.

A typical difficulty when designing scRNA-seq experiments are technical artifacts such as batch effects, which can then cause a covariance shift in the data. Since the investigation of batch correction methods is outside our manuscript’s scope, and they have already been described in detail in other sources^27–29^, we omit a detailed description here. Consequently, we examined all models without using potentially available batch correction capabilities. However, we expect that researchers could apply batch correction methods to full-scale experiment data after designing a study based on data generated by deep generative models. Currently, batch effects cannot be learned with scDBMs. In the future, however, scDBMs could be extended to include a batch correction component. We could take advantage of the flexibility of DBMs by developing a multimodal scDBM. In other application examples, this capability has already been investigated^30^. To be precise, we could train two input networks. One receives the scRNA-seq gene expression data as input, and another that contains information about batches or other technical effects. For example, information about batches could be one-hot-encoded, after which this information can be learned with a binary DBM and then linked in the deeper layers to that of the scRNA-seq input.

It has proven difficult to evaluate the quality of synthetic data from generative models^9^. One drawback is the sometimes ambiguous examination of synthetic data based on multiple quality measures. We are currently working on a method to evaluate the quality of latent representations in generative models using statistical tests based on resampling null distributions. This approach could then lead to a simplification of designing scRNA-seq experiments using deep generative models, as we could directly infer the statistical power.

Overall, it is a great challenge to infer from a few observations to larger datasets and, depending on the field of application, to monitor the corresponding quality characteristics. Models that specifically target the discovery of rare events could likely provide further performance improvements. Finally, we are confident that deep generative models have great potential for generating synthetic datasets. In particular, these methods could mean an improvement in the planning of future experiments.

## Methods

### Single-cell variational inference

Lopez et al.^7^ proposed a method called single-cell variational inference (scVI), which utilizes the structure of VAEs to encode the transcriptome onto a lower-dimensional representation from which the input is reconstructed. Just as the scDBM, scVI is also based on the (zero-inflated) negative binomial distribution^7^.

The model comprises two components, the encoder and the decoder parts of the network. Lopez et al.^7^ use four neural networks for encoding the size factors and the latent variables using the variational distribution $q(z_{n},l_{n} |x_{n},s_{n})$ as an approximation to the posterior $p(z_{n},l_{n} |x_{n},s_{n})$, where $z_{n}$ is a low-dimensional vector of Gaussians, $l_{n}$ is a one-dimensional Gaussian encoding technological differences in capture efficiency and sequencing depth, $x_{n}$ is the vector of observed expressions of all genes of cell *n*, and $s_{n}$ describes the batch annotation for each cell^7^. The variational distribution can be written as:

1 $$\begin{aligned} q(z_{n},l_{n} | x_{n}, s_{n}) = q(z_{n} | x_{n}, s_{n})q(l_{n} | x_{n}, s_{n}). \end{aligned}$$

Therefore, the variational lower bound is:

2 $$\begin{aligned} \log p(x|s) \ge E_{q(z,l | x, s)} \log p(x|z,l,s) - D_{KL}(q(z|x,s)||p(z)) - D_{KL}(q(l|x,s)||p(l)). \end{aligned}$$

The probabilistic model of scVI is based on a gamma-Poisson mixture. It starts by sampling from the latent space, a standard multivariate normal distribution, which is then fed into a neural network—together with the batch annotation. The neural network then learns the mean proportion of transcripts expressed across all genes. The output is used to sample from a gamma distribution together with the inverse dispersion $\theta _{m}$. The model accounts for technical effects by incorporating a library size scaling factor which, in combination with the gamma-distributed samples, is used to sample from a Poisson distribution. This mixture of the gamma and Poisson distribution is equivalent to the negative binomial distribution^7^. scVI additionally learns a neural network to account for technical dropouts.

Observations are generated from the scVI approach by using original data as input and then sampling from the posterior distribution *p*(*z*|*x*). A straightforward approach for generating more samples than were used during training is to create (multiple) copies of the original data. For example, for scVI trained on 384 cells, we sampled from the model seven times and stacked the resulting samples together to make inference about a larger number of cells. As an alternative, we adapted scVI to sampling from the prior distribution *p*(*z*) instead of the more common sampling from the posterior *p*(*z*|*x*). To do that, we changed the inference procedure to sample latent *z* from *Normal*(0, 1) and library sizes from $Normal(l_{\mu },1)$.

### Single-cell deep Boltzmann machine

We adapted deep Boltzmann machines (DBMs), an unsupervised neural network approach with multiple hidden layers^3^, to the negative binomial distribution. Specifically, we employ the exponential family harmonium framework^31^ that allows restricted Boltzmann machines (RBMs), the single-hidden layer version of DBMs, to deal with any distribution from the exponential family as input. This framework was further extended and simplified by Li et al.^32^.

We use a parametrization of the negative binomial probability mass function that has been suggested by Risso et al.^33^:

3 $$\begin{aligned} p_{NB}(v;\mu ,\theta ) = \frac{\Gamma (v+\theta )}{\Gamma (v+1)\Gamma (\theta )}\bigg (\frac{\theta }{\theta + \mu }\bigg )^{\theta }\bigg (\frac{\mu }{\theta + \mu }\bigg )^{v}. \end{aligned}$$

The mean of the distribution is denoted as $\mu$, the variance is given by $\mu + \mu ^{2} / \theta$, and $\theta$ is the inverse dispersion. $\Gamma$ denotes the gamma function.

For simplicity, we describe a three-layer DBM where the visible layer corresponds to an input of unique molecular identifier (UMI) counts for M genes, which can be modeled by a negative binomial distribution^34^. The first and second hidden layers are denoted as $h^{(1)}$ and $h^{(2)}$, respectively.

Following Li et al.^32^, we define the energy function of the state $\{x,h^{(1)},h^{(2)}\}$ as:

4 $$\begin{aligned} E(x,h^{(1)},h^{(2)};\Theta )&= -a^{T}x - \sum _{m=1}^{M}\log \bigg ( \frac{(x_{m}+\theta _{m}-1)!}{(\theta _{m}-1)!x_{m}!} \bigg ) \nonumber \\&\quad - b^{(1)T}h^{(1)} - b^{(2)T}h^{(2)} - x^{T}W^{(1)}h^{(1)} - h^{(1)T}W^{(2)}h^{(2)}. \end{aligned}$$

Here, *a*, $b^{(1)}$, and $b^{(2)}$ are the bias terms of the first, second, and third layer, respectively. Furthermore, $W^{(1)}$ and $W^{(2)}$ denote the weight matrices connecting the layers. Hence, $\Theta =(\theta ,a,b^{(1)},b^{(2)},W^{(1)},W^{(2)})$ are the model parameters. Therefore, the probability of the visible vector is defined as:

5 $$\begin{aligned} p(x;\Theta ) = \frac{1}{Z(\Theta )} \sum _{h^{(1)},h^{(2)}} \exp (- E(x,h^{(1)},h^{(2)};\Theta )). \end{aligned}$$

$Z(\Theta )$ is the partition function which is typically intractable^3^. According to this, the conditional distributions over the visible and the two sets of hidden units are given as:

6 $$\begin{aligned} p(x|h^{(1)})&= \prod _{m=1}^{M} NB\bigg ({\hat{\mu }},{\hat{\theta }}\bigg ), \quad {\hat{\mu }} = \frac{{\hat{\theta }}_{m}e^{{\hat{a}}_{m}}}{(1 - e^{{\hat{a}}_{m}})} \end{aligned}$$

7 $$\begin{aligned} p(h^{(1)} | x,h^{(2)})&= \prod _{k=1}^{K} Bern\bigg (\sigma ({\hat{b}}^{(1)}_{k})\bigg ) \end{aligned}$$

8 $$\begin{aligned} p(h^{(2)} | h^{(1)})&= \prod _{l=1}^{L} Bern\bigg (\sigma ({\hat{b}}^{(2)}_{l})\bigg ), \end{aligned}$$

where ${\hat{a}}_{m} = a_{m} + \sum _{k=1}^{K} W_{mk}^{(1)} h_{k}^{(1)}$ represents the estimate for the visible bias of UMI counts per gene *m* ($m=1,\ldots ,M$) and the bias of the first and second hidden layer correspond to ${\hat{b}}^{(1)}_{k} = b^{(1)}_{k} + \sum _{m=1}^{M}W_{mk}^{(1)}x_{m} + \sum _{l=1}^{L}W_{kl}^{(2)}h_{k}^{(2)}$ and ${\hat{b}}^{(2)}_{l} = b^{(2)}_{l} + \sum _{k=1}^{K} W_{kl}^{(2)}h_{k}^{(1)}$, where $k = 1,\dotsc ,K$ and $l = 1,\dotsc ,L$ indicate the hidden nodes in the first and second hidden layer, respectively. The sigmoid activation function is denoted as $\sigma$, and *Bern*() indicates Bernoulli distributed random variables. Training of the scDBMs via stochastic gradient descent can be performed just as for standard DBMs. For a detailed description, see Salakhutdinov and Hinton^3,35^.

After training, synthetic observations can be generated by Gibbs sampling. It can be shown that Gibbs sampling produces asymptotically exact samples, which leads to more accurate results as compared to VAEs^16,36^. This comes at the cost of a higher computational burden, which might be acceptable in small sample scenarios. In contrast, scVI uses variational inference, which scales to scenarios with millions of observations but does not have the advantage of generating exact samples^16^.

#### Estimating the dispersion parameter

For the negative binomial distribution, we also need to determine values for the inverse dispersion parameter of each gene which is notoriously difficult^37^.

We use a regularized Fisher scoring algorithm^38^ to estimate the inverse dispersion parameter $\theta _{m}$ for each gene *m*. For this, we use the log-likelihood function of the negative binomial probability mass function (Eq. 3) indicated above. The Fisher scoring algorithm can be derived using a two-term Taylor expansion of the score function, the first derivative of the log-likelihood, at the initial choice of the inverse dispersion $\theta ^{0}_{m}$^39^. To stabilize estimates of the inverse dispersion parameters, we add $\frac{\lambda }{\theta ^{2}}$ as a regularization term to the log-likelihood, which results in the following scoring algorithm:

9 $$\begin{aligned} \theta _{m,k+1} = \theta _{m,k} + \frac{V(\theta _{m,k}) + \lambda \frac{2}{\theta ^{3}_{m,k}}}{{\mathcal {I}}(\theta _{m,k}) + \lambda \frac{6}{\theta ^{4}_{m,k}}}. \end{aligned}$$

Here, $V(\cdot )$ is the score function, ${\mathcal {I}}(\cdot )$ denotes the Fisher information matrix, $\lambda$ is the regularization parameter, and *k* is the current iteration step.

The inverse dispersion parameter $\theta _{m}$ corresponds to the amount of heterogeneity between cells, where a smaller $\theta _{m}$ indicates more heterogeneity. Recall that the negative binomial variance is defined as $\mu + \mu ^{2} / \theta$. Due to the regularization term in our model, smaller $\theta _{m}$ are subject to larger regularization. This ensures that we learn the baseline variability between cells, without deflating the estimates of the inverse dispersion due to, e.g., differences between clusters of cells or excess zeros.

#### scDBM training

By combining the scDBM with Fisher scoring, we can estimate all model parameters $\Theta =(\theta ,a,b^{(1)},b^{(2)},W^{(1)},W^{(2)})$. In the first step, we initialize all parameters at some reasonable values and learn only a subset of $\Theta$, namely, $(a,b^{(1)},b^{(2)},W^{(1)},W^{(2)})$. Hence, the inverse dispersion is fixed (Supplementary Fig. 6a). After a predefined number of epochs, say five, we use the regularized Fisher scoring algorithm to estimate the inverse dispersion parameter ${\hat{\theta }}_{m}$ and plug the new estimate into the scDBM. Accordingly, all parameters of the scDBM are refined after a fixed time, e.g., every five epochs (Supplementary Fig. 6b).

During training, biases and weights of the network have to be constrained, where $a_{m} = min\{ a_{m}, -\epsilon \}$ with $\epsilon = 10^{-10}$ and $w_{m,k} = min\{ w_{m,k},0\}$. This is done because we use the natural form of the exponential family and hence $a_{m}$ is used in logarithmic scale^32^.

### Evaluation of synthetic data quality

The overall approach taken here for evaluating the quality of generated synthetic observations is illustrated in Fig. 1. Specifically, a relatively large original dataset is used as ground truth data, and deep generative approaches are tasked with generating synthetic data based on pilot datasets drawn from the original data. We consider Seurat clustering^40,41^ on the UMAP representations^42^ of the original data as a typical data analysis workflow, which provides ground truth cluster labels for the original data. When subsequently assessing synthetic data, each generated observation is assigned the cluster label of the nearest original observation, as determined by Euclidean distance. If a generative approach can provide synthetic observations very close to the original observations, these cluster labels will correspond to a reasonable clustering solution also in the synthetic data. Thus, we can evaluate the quality of the synthetic data by calculating summary statistics for the clusters in the synthetic data, and compare them to cluster statistics from the original data.

Specifically, we use the Davies–Bouldin index (DBI)

10 $$\begin{aligned} DBI(C_{K}) = \frac{1}{K} \sum _{i=1}^{K} D_{i}, \end{aligned}$$

where

11 $$\begin{aligned} D_{i} = max_{j \ne i} R_{ij} \end{aligned}$$

with between-cluster similarity

12 $$\begin{aligned} R_{ij} = \frac{S_{i} - S_{j}}{M_{ij}} \quad , \quad i,j=1,\ldots ,K \end{aligned}$$

the distance between cluster centroids

13 $$\begin{aligned} M_{ij} = \big \Vert {\bar{x}}_{i} - {\bar{x}}_{j} \big \Vert _{p} \end{aligned}$$

and within-cluster dispersions

14 $$\begin{aligned} S_{k} = \Bigg ( \frac{1}{n_{k}} \sum _{c(i) = k} \bigg \Vert x_{i} - {\bar{x}}_{k}\bigg \Vert _{2}^{q}\Bigg )^{\frac{1}{q}}, \end{aligned}$$

where we set $p=q=2$. Consequently, a small DBI indicates homogeneous and well-separated clusters^43,44^.

Moreover, we consider the adjusted Rand index (ARI). The ARI provides a similarity metric between two cluster groupings.

15 $$\begin{aligned} ARI = \frac{\sum _{ij} {n_{ij}\atopwithdelims ()2} - \left[ \sum _{i} {a_{i}\atopwithdelims ()2} \sum _{j} {b_{j}\atopwithdelims ()2} \right] / {n \atopwithdelims ()2}}{(1/2) \left[ \sum _{i} {a_{i}\atopwithdelims ()2} + \sum _{j} {b_{j}\atopwithdelims ()2} \right] - \left[ \sum _{i} {a_{i}\atopwithdelims ()2} \sum _{j} {b_{j}\atopwithdelims ()2} \right] /{n \atopwithdelims ()2}}, \end{aligned}$$

where $n_{ij}$ denotes the number of objects that the two cluster partitions have in common and $a_{i}$ and $b_{j}$ correspond to the respective sums in the corresponding contingency table^44^.

To examine whether the models learn to adequately represent frequencies of different cell types, we also compare the number of cells per cluster in the original data and the synthetic observations.

It should be noted that an in-depth evaluation of samples, instead of comparing model fit based on the log-likelihood, is indispensable because it was shown that comparing deep generative models based only on the log-likelihood can be misleading. In particular, even when log-likelihood is low, the quality of generated samples can be good and vice-versa^9^. In contrast, we focus on properties, such as cluster quality, which are important for experimental design.

### Tuning deep generative models

Since we want to investigate the quality of the generated data with the least possible influence of the hyperparameters of the individual models, we keep the architectures of the networks rather simple. The learning rate of the scDBM has to be set relatively small. This is because the reconstruction error can get very large for high expression values. Hence, the corresponding weights will get a very big learning signal^45^. It follows that we also slightly increased the number of epochs. Other than that, we keep the hyperparameter settings largely the same for all models (Supplementary Table 1). We tuned the hyperparameters of the respective models only once per dataset. Hence, we trained the models independently of the size of the dataset. In reality, we would of course tune the networks explicitly for the dataset at hand and most likely achieve better performance. However, tuning each model by hand would be unfeasable in this setting (30 sub-samples, 6 dataset sizes, 3 models—540 trainings in total). We split the data into random train and test subsets with 0.7 being the proportion of the data included in the trainingset.

For scVI, we used the default ReLU activation functions for hidden layers and the sigmoid activation function for hidden layers in scDBMs. We stick to the default parameters for the architecture of scVI. Therefore we use one hidden layer for both the encoder and the decoder network. Furthermore, the dimensionality of the latent space defaults to 10. We also chose two hidden layers for the scDBM and set the dimensionality of the latent space between two to four, depending on the dataset.

### Data description and processing

We evaluate the performance of the two scVI variants and the scDBM approach on four diverse datasets. First, a 10$\times$ Genomics dataset containing peripheral blood mononuclear cells from a healthy donor is considered^46^. We preprocessed the data following Amezquita et al.^47^, after which 4182 cells and 1000 highly variable genes were left for downstream analysis. We refer to this dataset as *PBMC*4*k* throughout this work.

For comparison, we use a Smart-seq2 dataset of human pancreatic cells from multiple donors throughout the manuscript^48^. We also preprocessed the data according to the approach in Amezquita et al.^47^. Based on this, we extracted the 2000 most highly variable genes leaving 2090 cells for analyses. Within this manuscript, we refer to this dataset as *Segerstolpe*.

Third, analyses are performed on a dataset of neuronal subtypes in the mouse cortex and hippocampus, where Zeisel et al.^49^ sequenced 3005 cells from male and female juvenile mice. We specifically consider data from 2816 cells and 1816 highly variable genes which were left after preprocessing^47^. We refer to this dataset as *Zeisel* throughout this work.

Additionally, we demonstrate the performance on a currently unpublished scRNA-seq dataset from the hippocampus of three embryonic (E16.5) mice processed with the CEL-Seq2 protocol^50,51^. The unnormalized count matrix contained 3808 cells, and we selected the 1500 most highly variable genes for downstream analysis. We used scran and scater^52,53^ for pre-processing. We refer to this dataset as *Hippocampus*4*k* throughout this work. The results for *Zeisel* and *Hippocampus*4*k* can be found in the Supplementary Information.

### Implementation

The scDBM implementation is based on the Julia package ‘BoltzmannMachines.jl’^54^ and extends the packages’ scope to scRNA-seq data which is available at https://github.com/MTreppner/scDBM.jl. The code to reproduce all analyses and figures can be found on the following Github repository: https://github.com/MTreppner/scDBM-paper.

Furthermore, we used the Python implementation of scVI (https://github.com/YosefLab/scVI), which we adapted to be able to sample from the prior distribution.

## Supplementary Information

Supplementary Information.
