# Supplementary material for: Synthetic single cell RNA sequencing data from small pilot studies using deep generative models
Source: Sci Rep. 2021 Apr 30;11:9403. doi: 10.1038/s41598-021-88875-4 (PMC8087667; doi:10.1038/s41598-021-88875-4)
Supplement: Supplementary file 1 — Supplementary Information. [file 41598_2021_88875_MOESM1_ESM.pdf]

Synthetic Single Cell RNA Sequencing Data from Small Pilot Studies using Deep Generative Models

Martin Treppner[1,2,4,*], Adrián Salas-Bastos[3,4], Moritz Hess[1,2], Stefan Lenz[1,2], Tanja Vogel[3,5,6], and Harald Binder[1,2]

[1]Institute of Medical Biometry and Statistics, Faculty of Medicine and Medical Center - University of Freiburg, Freiburg, 79104, Germany
[2]Freiburg Center for Data Analysis and Modeling, University of Freiburg, Freiburg, 79104, Germany
[3]Institute of Anatomy and Cell Biology, Department of Molecular Embryology, Medical Faculty, University of Freiburg, Freiburg, 79104, Germany
[4]Faculty of Biology, University of Freiburg, Freiburg, Germany
[5]Center for Basics in NeuroModulation (NeuroModul Basics), University of Freiburg, Freiburg, 79104, Germany
[6]Freiburg Institute for Advanced Studies (FRIAS), University of Freiburg, Germany
[*]treppner@imbi.uni-freiburg.de

ABSTRACT

Deep generative models, such as variational autoencoders (VAEs) or deep Boltzmann machines (DBM), can generate an arbitrary number of synthetic observations after being trained on an initial set of samples. This has mainly been investigated for imaging data but could also be useful for single-cell transcriptomics (scRNA-seq). A small pilot study could be used for planning a full-scale experiment by investigating planned analysis strategies on synthetic data with different sample sizes. It is unclear whether synthetic observations generated based on a small scRNA-seq dataset reflect the properties relevant for subsequent data analysis steps.

We specifically investigated two deep generative modeling approaches, VAEs and DBMs. First, we considered single-cell variational inference (scVI) in two variants, generating samples from the posterior distribution, the standard approach, or the prior distribution. Second, we propose single-cell deep Boltzmann machines (scDBM). When considering the similarity of clustering results on synthetic data to ground-truth clustering, we find that the $scVI_{posterior}$ variant resulted in high variability, most likely due to amplifying artifacts of small datasets. All approaches showed mixed results for cell types with different abundance by overrepresenting highly abundant cell types and missing less abundant cell types. With increasing pilot dataset sizes, the proportions of the cells in each cluster became more similar to that of ground-truth data. We also showed that all approaches learn the univariate distribution of most genes, but problems occurred with bimodality. Across all analyses, in comparing 10x Genomics and Smart-seq2 technologies, we could show that for 10x datasets, which have higher sparsity, it is more challenging to make inference from small to larger datasets. Overall, the results show that generative deep learning approaches might be valuable for supporting the design of scRNA-seq experiments.

Supplementary Information

	PBMC4k		Zeisel		Hippocampus4k		Segerstolpe	
Model	scDBM	scVI	scDBM	scVI	scDBM	scVI	scDBM	scVI
Learningrate	0.00005	0.001	0.00001	0.001	0.00001	0.001	$1.0e^{-11}$	0.01
Hidden layers	2	2	2	2	2	2	2	2
Epochs	120	100	200	100	200	100	160	150

Supplementary Table 1. Hyperparameters

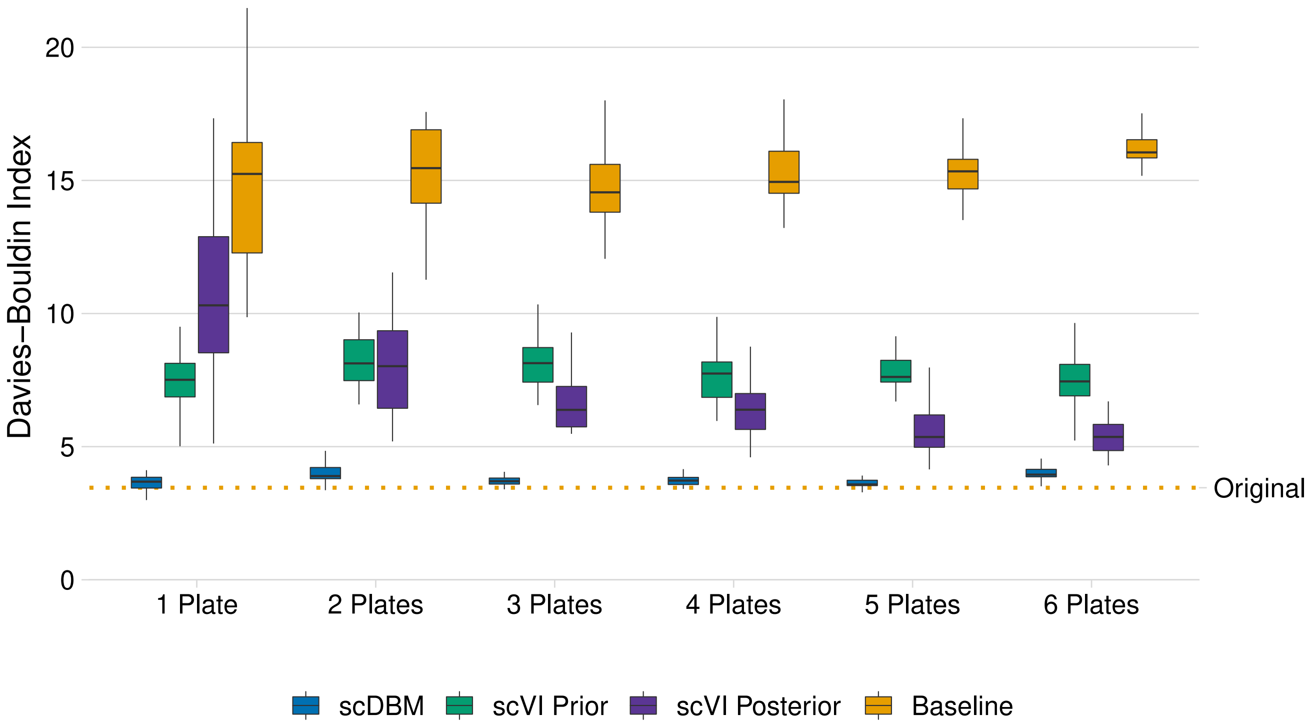

Supplementary Figure 1. Davies-Bouldin index (DBI), indicating the quality of synthetic data generated by scDBM, scVI (prior and posterior sampling), and a baseline from pilot data of different sizes (*Zeisel*). Each boxplot represents 30 sub-samples from the original data (lower and upper hinges correspond to the 25th and 75th percentiles). The orange line indicates the reference DBI for the Seurat clustering on the original data (*Zeisel*) with 2816 cells.

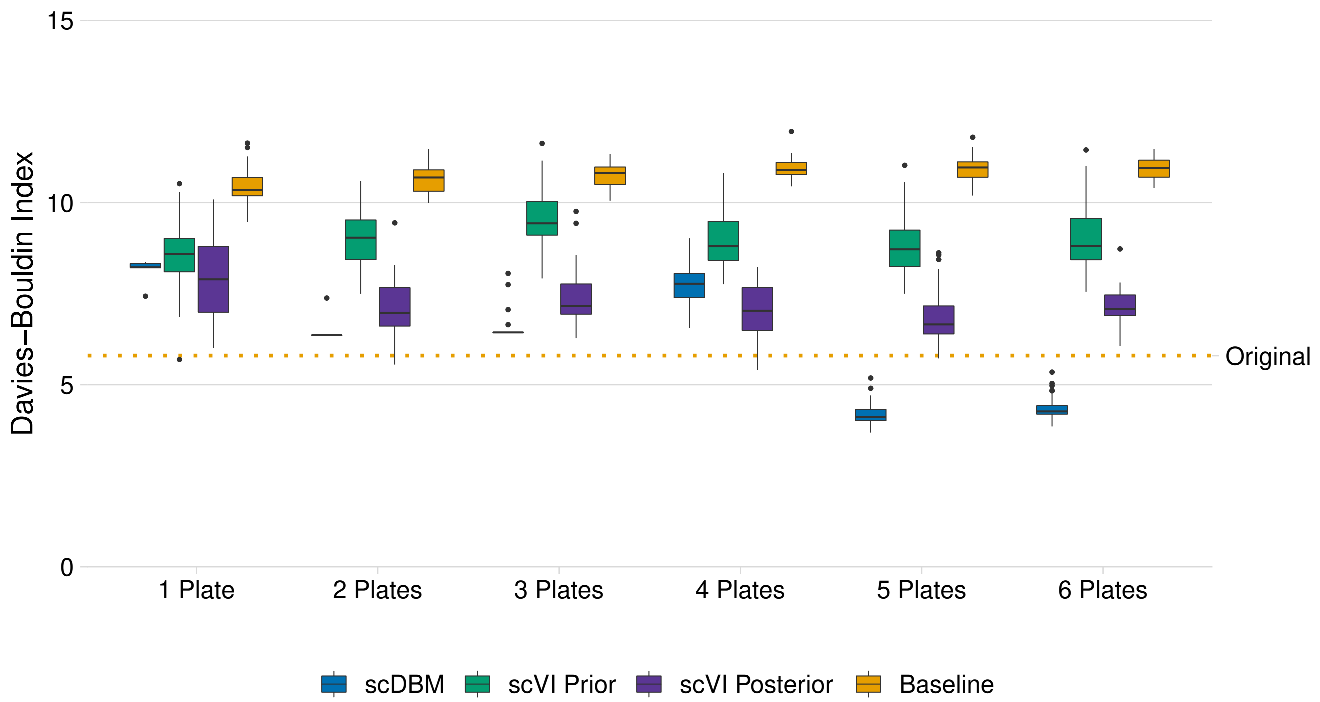

Supplementary Figure 2. Davies-Bouldin index (DBI), indicating the quality of synthetic data generated by scDBM, scVI (prior and posterior sampling), and a baseline from pilot data of different sizes (Hippocampus4k). Each boxplot represents 30 sub-samples from the original data (lower and upper hinges correspond to the 25th and 75th percentiles). The orange line indicates the reference DBI for the Seurat clustering on the original data with 3808 cells.

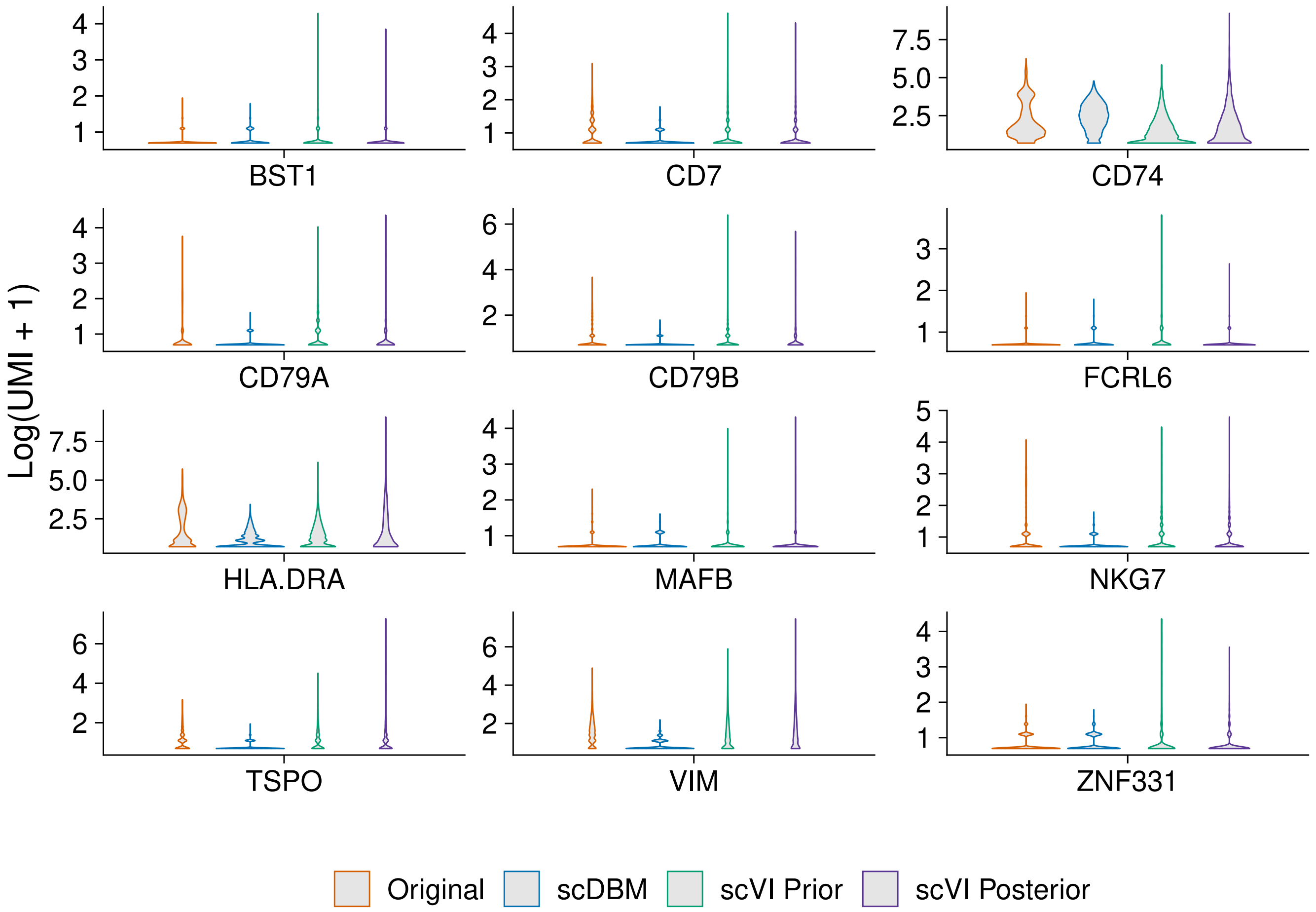

Supplementary Figure 3. Univariate distributions of expression values for exemplary genes, as generated by scDBM and scVI when trained on 384 cell pilot data sub-sampled from the *PBMC4k* dataset, compared to the original data.

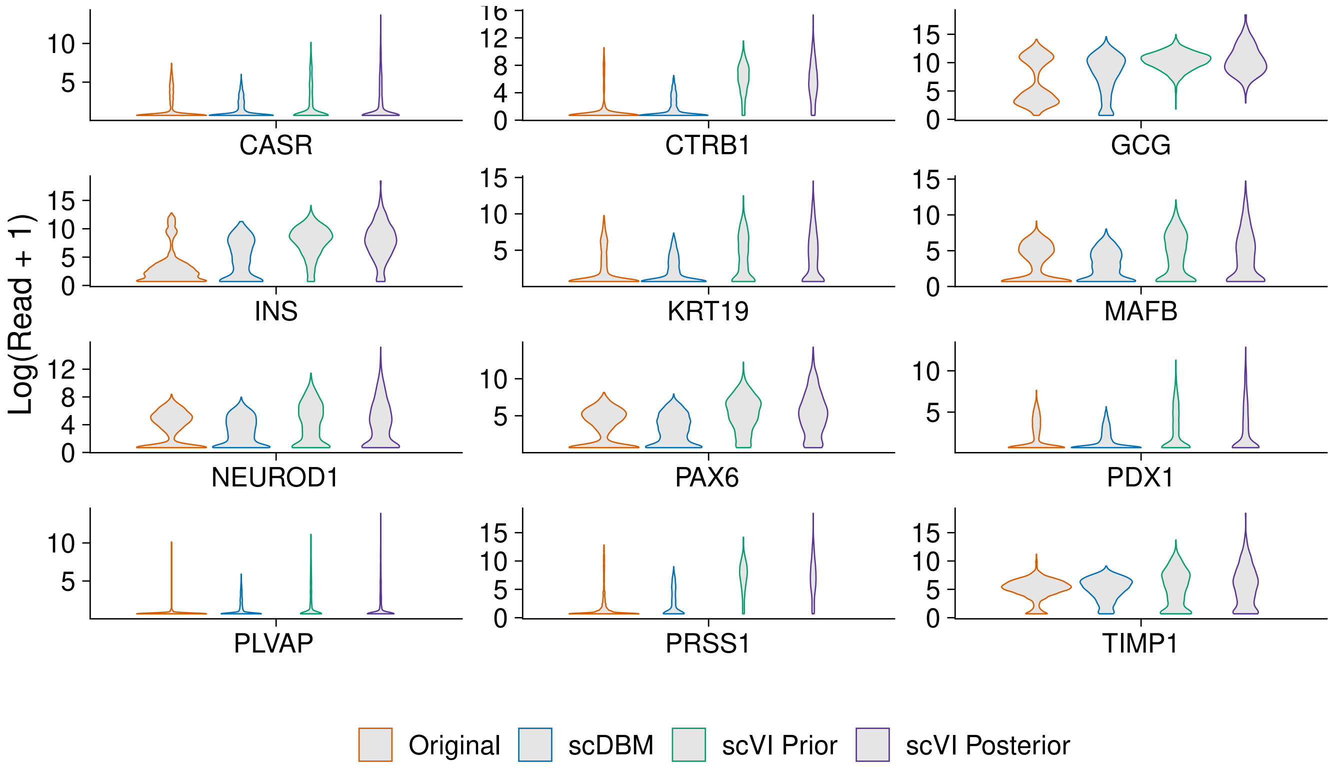

Supplementary Figure 4. Univariate distributions of expression values for exemplary genes, as generated by scDBM and scVI when trained on 384 cell pilot data sub-sampled from the *Segerstolpe* dataset, compared to the original data.

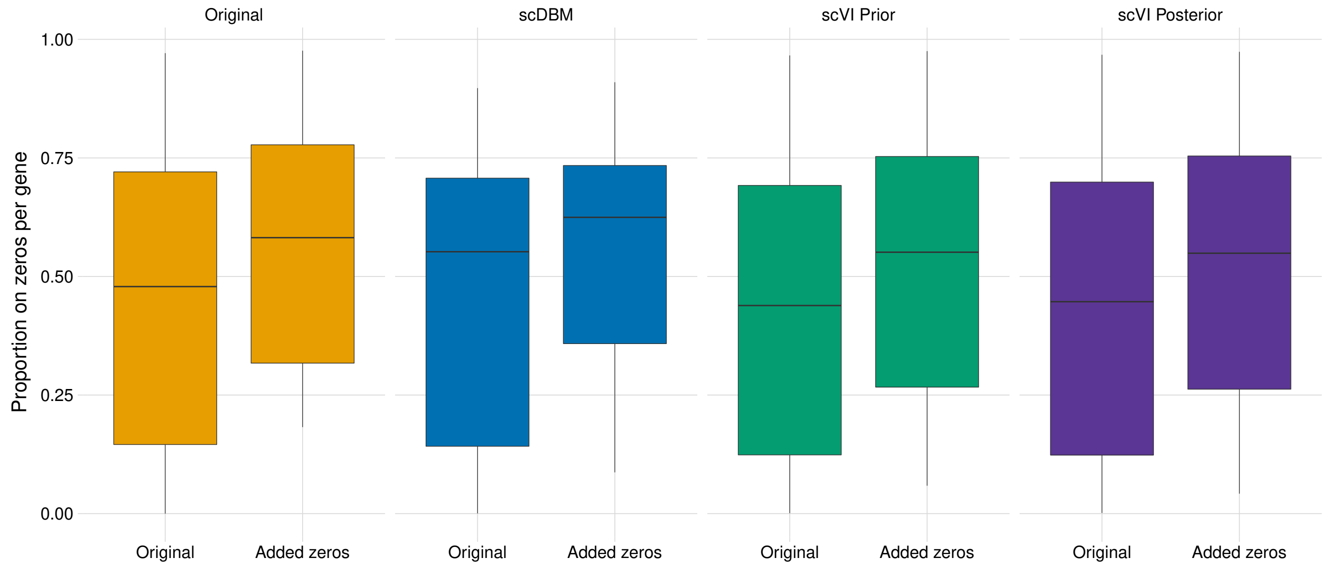

Supplementary Figure 5. Proportion of zeros per gene for the original *Segerstolpe* dataset and the same dataset with 20% of gene expression counts set to zero across all models.

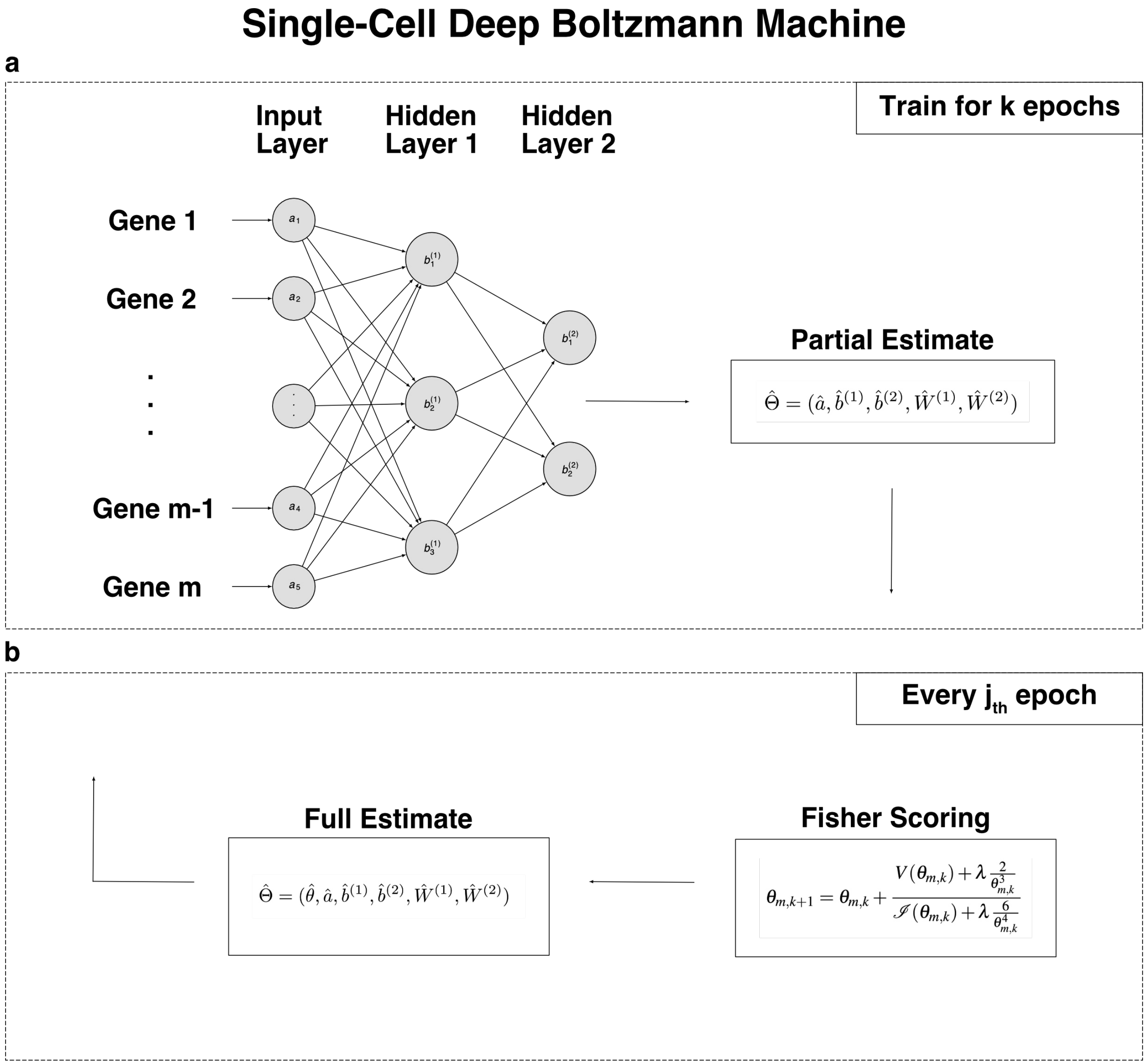

Supplementary Figure 6. Single-cell deep Boltzmann machine (scDBM) network structure (**a**) and iterative estimation procedure (**b**).

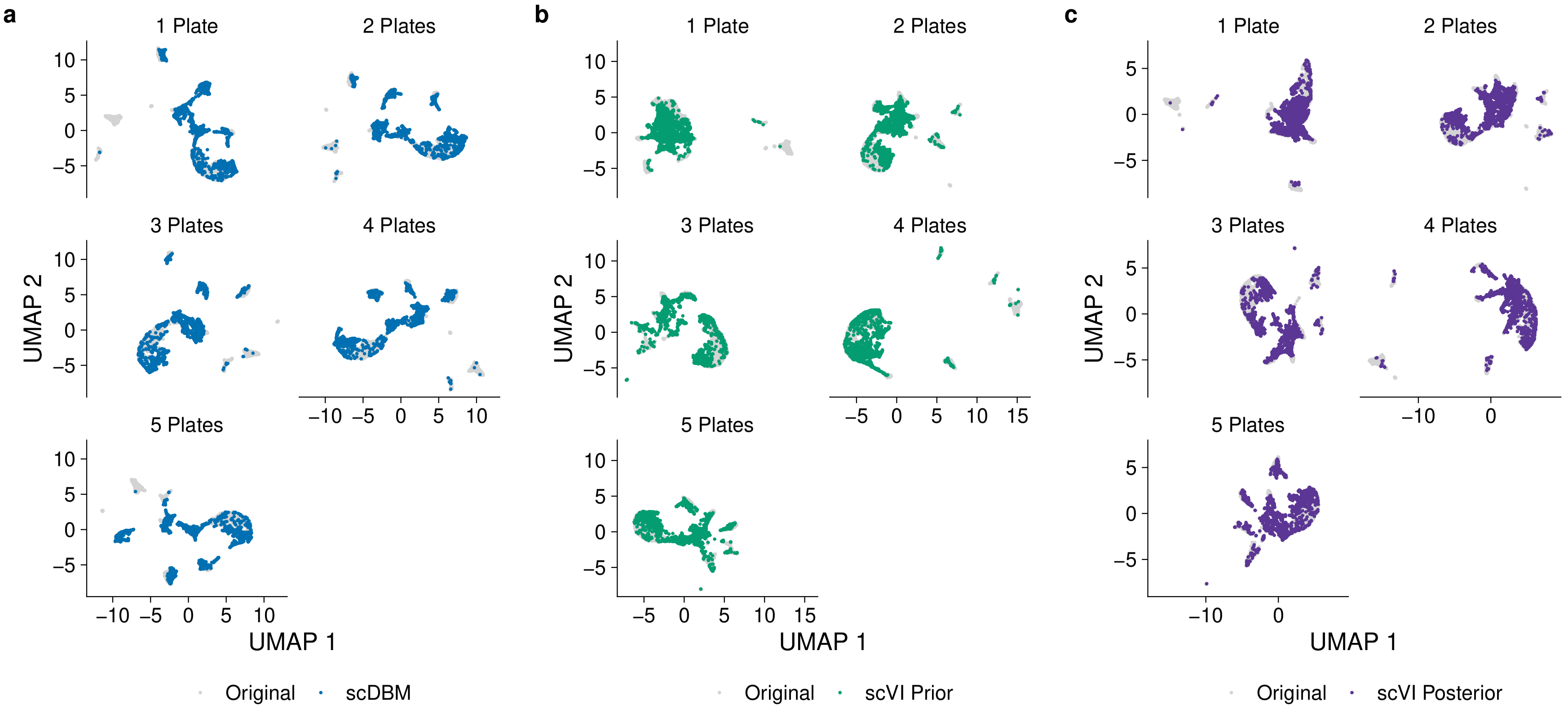

Supplementary Figure 7. Exemplary UMAP representations for the *Segerstolpe* dataset across all models and all sample sizes.
